# Serum PCB levels and congener profiles among US construction workers

**DOI:** 10.1186/1476-069X-6-25

**Published:** 2007-08-31

**Authors:** Robert F Herrick, John D Meeker, Russ Hauser, Larisa Altshul, George A Weymouth

**Affiliations:** 1 Harvard University School of Public Health, Huntington Ave, Boston MA, USA; 2 University of Michigan School of Public Health, Observatory St, Ann Arbor, MI, USA; 3 International Union of Bricklayers and Allied Craft Workers, (Retired), Local 3, Boston, MA, USA

## Abstract

**Background:**

The presence of PCB in caulking (sealant) material found in masonry buildings has been well-documented in several countries. A recent investigation of 24 buildings in the greater Boston area found that 8 buildings had high PCB levels in caulking materials used around window frames and in joints between masonry blocks. Workers removing caulking material have been shown to have elevated serum PCB levels.

**Methods:**

This project compared serum PCB levels among male workers who installed and/or removed PCB-containing caulking material from buildings in the greater Boston area with reference serum PCB levels from 358 men from the same area. Serum PCB levels were measured in the same laboratory by liquid-liquid extraction, column chromatography clean-up and dual capillary column GC/microECD analysis.

**Results:**

When the congener profiles were compared between the reference population and the construction workers, the serum levels of the more volatile, lighter PCBs (di-, tri-and tetrachloro, sum of IUPAC# 6–74) were substantially higher among the construction workers. One of the youngest workers had the lowest total serum PCB levels (sum of 57 congeners) of all 6 workers, but the contribution of more volatile (less chlorinated) PCB congeners (#16, 26,28,33,74,66, and 60) was markedly higher than in other 5 workers and reference men. Only this worker was working on a job that involved removing PCB caulking at the time of the blood sampling.

**Conclusion:**

While the results of this pilot study are based upon small numbers (6 construction workers who handled PCB caulking), the serum PCB levels among the construction workers exceed the referents. Comparison of the congener profiles suggests that there are substantial differences between the construction workers and the general population samples. These differences, and the similarities of profiles among the construction workers strongly suggest that occupational contact with caulking material can be a major source of PCB exposure for construction workers.

## Background

Polychlorinated biphenyls (PCBs) are organic chemicals that have been associated with a wide range of adverse health effects among those exposed. PCBs are currently classified as probable human carcinogens, and are also toxic to the immune, reproductive, nervous, and endocrine systems [1]. Since PCBs also persist and accumulate in the environment, the US Environmental Protection Agency (EPA) banned the manufacture of PCBs in 1977 and strictly regulates the storage, transport, and disposal of PCB materials [2].

While PCBs were primarily used in electrical equipment, they were also used in building materials such as caulking (also described as sealants). Buildings that were constructed or refurbished prior to 1977 may still contain caulking with elevated levels of PCBs. Because there are generally no requirements that building materials be tested for PCBs, caulking is rarely analyzed for PCB content and is poorly recognized as a potential hazard.

In September 2003, a preliminary survey was conducted to assess the extent of PCB contamination in existing structures. Caulking samples (approximately 10 grams each) were collected from 24 buildings in the Greater Boston area and analyzed for PCBs (EPA Method SW846 8082A). A retired construction tradesman (PCC, for pointer, caulker, cleaner specialist from the Bricklayers Union, Local 3, Boston) selected buildings where caulking was likely to have been installed prior to 1977. These buildings included schools, university housing, museums, and office buildings. This survey revealed that 8 of the 24 buildings contained caulking with PCB concentrations greater than the EPA limit of 50 ppm. In these eight samples, PCB concentrations ranged from 70.5 to 36,200 ppm, median of 7,990 ppm and mean of 15,654 ppm. EPA considers materials exceeding PCB content of 50 ppm that were not specifically authorized for use by EPA to be "unauthorized use" non-liquid PCB products that require removal and decontamination [2]. The PCB concentrations found in the caulking were hundreds of times greater than the EPA limit [3].

As this material is now nearly 30 years old, it is reaching the end of its useful life and is deteriorating in many cases. Construction workers are currently removing old PCB-containing caulking from many buildings using manual and power tools. The caulking can be removed in several ways, including cutting the bulk material from the masonry joints or around window perimeters by hand, using a fixed razor knifes, electric saws, or electric caulking cutters. Once the bulk caulking is removed, a high speed grinder with a diamond wheel or abrasive disk is used to grind away any caulking left on the surface. The grinding is performed to insure adhesion as the replacement caulking materials may be incompatible with the old caulking. Heat guns can be used in a case of historical renovation when removing hardened caulking so as not to mark or harm the surface in any way, to insure historical appearance. These removals are performed without testing the caulking to determine its PCB content, and workers generally do not use protective equipment. The caulking, and the surrounding masonry that is removed are disposed of as general demolition waste.

This project was designed to compare serum PCB levels among workers who reported installing and/or removing PCB-containing caulking material from buildings in the Greater Boston area to a reference group of Boston area men.

## Methods

A convenience sample of construction workers likely exposed to PCB-containing caulking was recruited from current and retired members of a local labor union. In addition to collecting a blood sample, the workers were interviewed about their work history and work with PCB-containing caulk. They were asked about their construction work activities, specifically whether they had ever installed the PCB-containing caulk (which was banned in 1977). Workers who were currently employed (not retired) were also asked about their recent work activities prior to the time of our sample collection (February 2005). The reference blood PCB levels [4] were from 358 men between the ages of 18 and 51 years who were seeking infertility diagnosis from the Vincent Burnham Andrology lab at Massachusetts General Hospital in Boston (January 2000 – May 2003).

A nonfasting venous blood sample was collected from the six construction workers in March 2005. Blood samples were centrifuged and serum stored in glass wheaton vials at -80 °C until analysis. Measurement of PCBs, p,p'-DDE and HCB in serum was conducted by the Organic Chemistry Analytical Laboratory, Harvard School of Public Health, Boston, MA. Serum extracts were analyzed by dual capillary column gas chromatography with electron capture detection (GC/micro ECD) and quantified based on the response factors of individual PCB congeners relative to an internal standard [4]. Target analytes included 57 individual PCB congeners, p,p'-DDE, and HCB. We have reported wet weight serum PCB concentrations (e.g. ng/g serum), however serum lipid levels are also reported for the six subjects so lipid-standardized PCB can be estimated for comparison with other studies since most investigators to date have used a lipid-standardized approach. Recent evidence suggests that bias can be introduced depending upon the method used to account for serum lipid content when assessing associations between serum PCB concentrations and health endpoints. The use of wet weight serum PCB concentrations (e.g. ng/g serum) in multivariate regression models with adjustment for serum lipids as a covariate was shown to be least prone to bias in a large simulation study [5].

## Results

The characteristics of the six construction workers who participated in the study are presented in Table 1. They ranged in age from 36 to 72 years, and two were retired. Four of the workers recalled installing PCB-containing caulk before about 1980, and all had done work removing it. The reference subjects were men of similar age (mean 36, SD 5.3, range 18–51) who were primarily residents of the greater Boston area.

**Table 1:** Characteristics of construction worker study subjects and serum PCB levels (Σ 57 congeners)

ID#	Birth Year	Age	Retired Y/N	Years worked	Current job	Ever installed PCB caulk	Time since last PCB work	Sum PCB ng/g serum	Ref* PCB ng/g serum	Serum lipids (%)
1	1945	60	Y	36	Retired PCC	Y	2 years	1.86	1.64	0.12
2	1954	51	N	30	Water proofer	Y	Approx. 1 month	2.11	1.64	0.24
3	1965	40	N	20	PCC	N	Currently working w/PCB	1.05	1.14	0.35
4	1969	36	N	16	Water proofer	N	Approx. 1 month	1.51	1.14	0.91
5	1939	66	N	45	Mason	Y	Approx. 1 month	1.77	1.64	0.83
6	1933	72	Y	54	Retired PCC	Y	1 year	8.70	1.64	0.58

*Reference PCB values are means for 5-year age groups from 358 men in the Greater Boston area. Referent subjects range from 18–51 years old.

### Referents compared to construction workers

**Figure 1:**
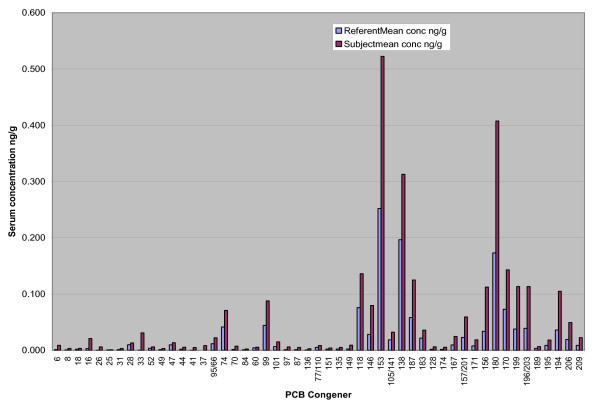
Referent and Worker Mean Serum Level 57 PCB Congeners. The contrast in the congener composition by concentration reflects the influence of worker #6, who was the oldest, and had the longest duration of work with PCB caulking.

**Figure 2:**
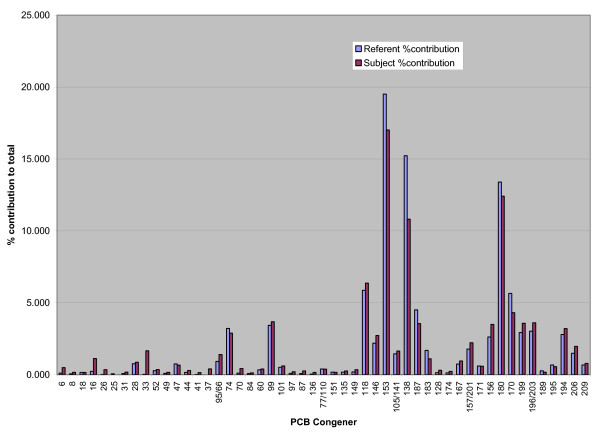
Referent and Worker Percent Contribution 57 PCB Congeners. The contrast in congener composition by percent contribution is apparent as the contribution of lighter congeners (di, tri-, and tetra-chlorinated, PCB 6–74) to the total PCB level is 11.7 % for the construction workers, compared to 6.5% for the referents.

Serum concentrations for the referents and construction workers were highest for congeners 153, 138, 180, 118, and 170 (Figure 1). These five congeners comprised 59.6% of the total PCB serum concentrations for the 358 reference men and 50.9% for the 6 construction workers (Figure 2, Table 2). When the congener profiles were compared between the reference population and the construction workers, the serum levels of the lighter PCBs (di-, tri- and tetrachloro, sum of IUPAC# 6–74) were substantially higher among the construction workers. For these 6 subjects, the mean level of the lighter congeners was 0.23 ng/g serum, SD 0.08, range 0.15–0.38 ng/g compared to a mean of 0.09 ng/g, SD 0.06, range 0.02–0.62 ng/g for the reference population (Table 3). We did not test for statistically significant differences between datasets since the construction workers' dataset was small (n = 6). Instead, we presented results in figures and tables to highlight the differences.

**Table 2:** Subject and referent mean serum concentrations and percent contribution by PCB congener

PCB Congener	Referent Mean conc ng/g	Subject Mean conc ng/g	Referent %contribution	Subject %contribution
6	0.001	0.009	0.108	0.491
8	0.001	0.004	0.060	0.166
18	0.002	0.003	0.158	0.160
16	0.003	0.021	0.234	1.123
26	0.000	0.006	0.018	0.354
25	0.001	0.001	0.066	0.001
31	0.001	0.003	0.082	0.180
28	0.010	0.013	0.766	0.867
33	0.000	0.031	0.024	1.668
52	0.004	0.006	0.277	0.357
49	0.001	0.003	0.098	0.165
47	0.010	0.014	0.749	0.667
44	0.002	0.006	0.158	0.292
41	0.000	0.005	0.012	0.153
37	0.000	0.008	0.003	0.409
95/66	0.012	0.022	0.922	1.407
74	0.042	0.071	3.217	2.893
70	0.001	0.008	0.111	0.427
84	0.001	0.002	0.079	0.128
60	0.004	0.006	0.339	0.402
99	0.044	0.088	3.436	3.680
101	0.007	0.015	0.516	0.613
97	0.001	0.006	0.086	0.206
87	0.001	0.005	0.082	0.266
136	0.001	0.003	0.040	0.162
77/110	0.005	0.009	0.399	0.388
151	0.002	0.004	0.180	0.167
135	0.002	0.005	0.184	0.262
149	0.003	0.009	0.194	0.351
118	0.076	0.136	5.871	6.368
146	0.028	0.080	2.192	2.726
153	0.252	0.522	19.510	17.017
105/141	0.019	0.032	1.451	1.649
138	0.197	0.313	15.224	10.814
187	0.058	0.125	4.510	3.557
183	0.022	0.036	1.693	1.108
128	0.002	0.006	0.143	0.311
174	0.002	0.005	0.129	0.230
167	0.010	0.025	0.743	0.957
157/201/177	0.023	0.594	1.783	2.225
171	0.008	0.019	0.605	0.592
156	0.034	0.112	2.621	3.501
180	0.173	0.408	13.394	12.412
170	0.073	0.143	5.649	4.316
199	0.038	0.113	2.938	3.582
196/203	0.039	0.114	3.031	3.614
189	0.004	0.007	0.271	0.177
195	0.009	0.018	0.677	0.558
194	0.036	0.105	2.802	3.208
206	0.019	0.049	1.489	1.973
209	0.009	0.023	0.676	0.792

**Table 3:** Light and heavy congener serum concentrations and % contributions to total PCB

PCBs	subject 1	subject 2	subject 3	subject 4	subject 5	subject 6	subject mean	ref** mean	subject median	ref Median
Serum concentration (ng/g)										

Light *	0.201	0.191	0.261	0.151	0.195	0.378	0.230	0.094	0.196	0.064
Heavy	1.66	1.91	0.788	1.36	1.57	8.33	2.61	1.19	1.80	0.949

Percent contribution										

Light	10.8	9.08	24.9	10.0	11.0	4.34	11.7	6.54	9.82	6.320
Heavy	89.2	90.9	75.1	90.0	89.0	95.7	88.4	91.7	90.17	93.68

*Light is comprised of the sum of congeners 6–74; Heavy is comprised of the sum of congeners 84 to 209

** Reference PCB values are means for 5-year age groups from 358 men in the Greater Boston area. Referent subjects range from 18–51 years old.

Levels of the heavy congeners (pentachloro and higher, sum of congeners 84 to 209) had a mean of 2.61 ng/g, SD 2.83, range 0.79–8.33 ng/g in workers compared to a reference mean of 1.19 ng/g, SD 0.83, range 0.16–9.47 ng/g (Table 3). When compared by percent contribution of the lighter congeners to the total PCB level, the construction worker mean contribution was almost double that of the referents, 11.7 % compared to 6.5% (Figure 2, Table 2).

**Figure 3:**
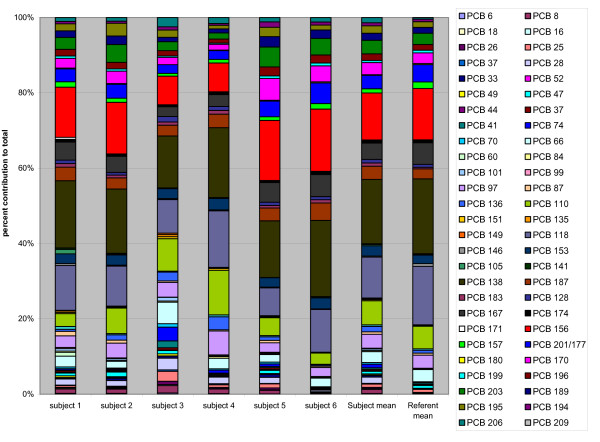
PCB Congener Profiles. Comparison of congener percent contribution by individual worker compared to referent mean.

When the congener profiles were examined by percent contribution to each subject's total PCB serum level, differences between the construction workers and reference group means were apparent. For the 6 construction workers, approximately 25% of the total PCB is contributed by congeners number 118 and lighter, while these congeners comprise only about 18% for the referents (Figure 3).

**Figure 4:**
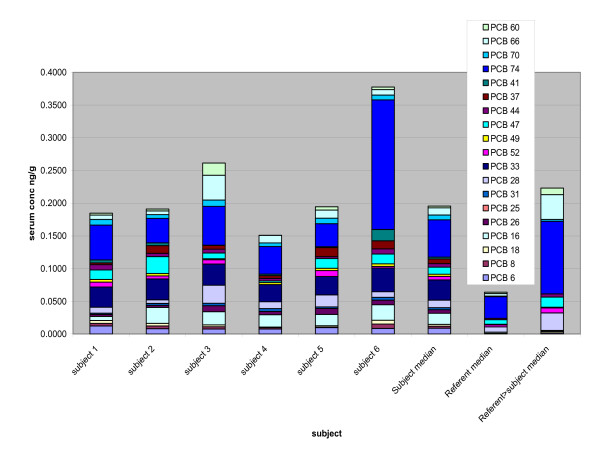
Serum Concentration Light Congeners. Comparison of congener concentrations by individual worker compared to referent mean for PCB congeners 6–74 (di, tri-, and tetra-chlorinated)

**Figure 5:**
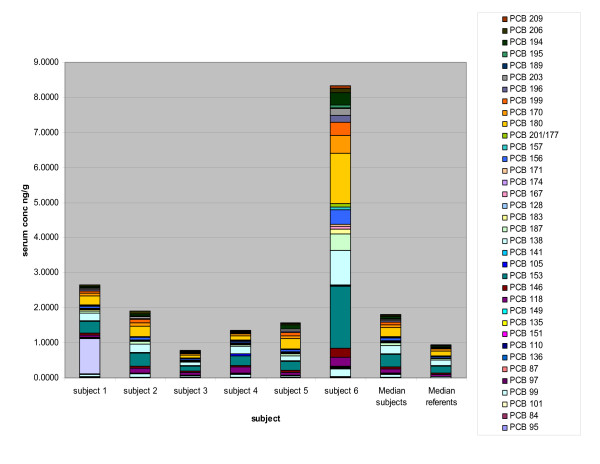
Serum Concentration Heavy Congeners. Comparison of congener concentrations by individual worker compared to referent mean for PCB congeners 84–209 (penta-chlorinated and higher)

When the serum concentrations of light vs. heavy congeners are examined by the specific PCBs comprising each group, differences in the composition among the six construction workers, and between these six workers and the reference population can be seen (Figures 4 and 5). Worker mean serum levels exceeded the reference mean by a factor of 5 or more for PCB 6,16, 26, 33, 37, 41, 70, 97, and 136. In fact, PCB 6 was detectable in only 44% of the 358 samples from the reference population, while several other congeners {PCB 26 (18%), 33 (39%), 37 (5%), 41 (18%), 97 (49%) and 136 (15%)} were detectable in fewer than half the reference samples. These congeners were present at detectable concentrations in all the samples from the 6 construction workers (The Method Detection Limit values for all PCB congeners were below 0.05 ng/g, with most of the congeners below 0.01 ng/g).

**Figure 6:**
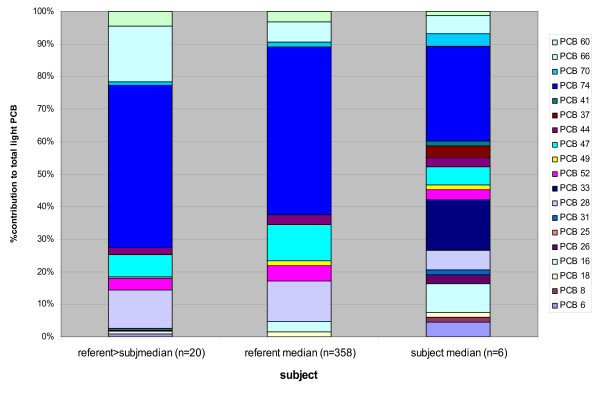
Light Congener % Contribution. Comparison of congener profiles for PCB 6-74 for the 20 referents exceeding the construction worker subject's median value for the sum of light congeners. These referents with the highest levels of light congeners have a very different profile of congeners compared to the construction workers, most notably for congeners 6, 8, 16, 26, 33, 37, and 41.

Of the 358 reference subjects, only 20 had serum levels of the light congeners (PCB 6–74) exceeding the construction worker subject's median value for the sum of light congeners (0.196 ng/g). Comparing these 20 reference subject congener profiles with the rest of the reference samples and the 6 construction workers, it is apparent that the congener profile of these referents with the highest levels of light congeners is very different from the construction workers (Figure 6). The difference is most notable for congeners 6, 8, 16, 26, 33, 37, and 41, which were nondetectable or present at very low levels (< 0.015 ng/g) in this subgroup of 20 referents. These 7 congeners comprised 60% of the sum of light congeners for the construction worker subjects. By contrast, the subgroup of referents (n = 20) had serum concentrations of these congeners that comprised only 27% of the light congener fraction, while this fraction for the entire (n = 358) referent group congeners was 38%.

### Comparison between workers

Subjects 3 and 6 exhibited the most striking differences in comparison to the referents, as well as to the other four construction workers. Subject 6 had the highest serum PCB level (8.72 ng/g). He was the oldest subject at the age of 72 and also had the longest duration of employment (54 years). He had worked as a PCC (pointer, cleaner, and caulker) tradesman for that entire time, and he reported that he had worked extensively in the installation of PCB caulking in the period of the mid-1950s to approximately 1980. He continued work in removing PCB caulking until 2004 when he retired, so he had not had contact with the caulking material for approximately 1 year prior to the time of the blood sampling in 2005. One of the youngest workers (subject 3) had the lowest total PCB levels of all construction workers, but the contribution of more volatile (less chlorinated) PCB congeners (#16, 26,28,33,74,66, and 60) was substantially higher than in the other 5 subjects and 358 reference men. Only this subject was working on a job that involved removing PCB caulking during the week immediately preceding the blood sampling (done on a Saturday), while the other three workers who were still employed (subjects 2, 4 and 5) had not been in contact with PCB caulk for at least one month prior to the time of blood sampling.

## Discussion

In a study of PCB sealant (caulking) removal from prefabricated concrete buildings in Finland, workers removing this material were found to be exposed to detectable airborne levels of PCBs 28, 52, 77, 101, 138, 153, and 180 [6]. The total PCB concentration (sum of 24 PCB congeners) in workers' sera ranged from 0.6 and 17.8 μg/l (mean 3.9 μg/l, median 1.9 μg/l). Population comparison values were 0.3–3.0 μg/l (mean 1.7 μg/l, median 1.5 μg/l) for the same 24 congeners. Inhalation appeared to be the primary route of occupational exposure, as there were strong associations between breathing zone inhalation PCB exposures and serum levels for PCB 28 (*r *= 0.70, *P *= 0.16) and PCB 52 and (*r *= 0.80, *P *= 0.06), despite the use of exhaust ventilated tools and protective equipment including respirators, gloves, and coveralls. Wingfors et al. [7] collected blood samples from workers (n = 36) involved in removing PCB sealants, as well as age- and sex-matched controls (n = 33). The samples were analyzed for 19 PCBs (congeners 28, 52, 47, 44, 74, 70, 66, 56/60, 95, 101, 99, 87, 110, 118, 105, 153, 138/163, 187, 180, 153). The levels (Σ 19 congeners) in the exposed workers were twice as high as those in the controls, with geometric means of 575 ng/g lipid compared to 267 ng/g for the controls (approximately 2.9 ng/g serum compared to 1.3 ng/g, assuming serum lipid content of 0.5%). The PCB congener patterns also differed between the workers and the controls, with much higher levels of many less chlorinated PCBs in the exposed workers, compared to the controls. Using principal component analysis, Wingfors concluded that PCBs 56/60, 66, 44, 70, and 110 were good markers for occupational exposures, while 153 and 180 reflected background (dietary) exposure.

The results of our examination of the 6 construction workers are generally consistent with the earlier findings for the workers conducting similar activities (removing PCB caulking/sealants) in Finland and Sweden. Overall PCB serum levels in our six workers were more similar to the referents than was reported in the other studies, possibly because the subjects in Finland and Sweden were working more extensively in removing the PCB-containing caulk (sealant). Because our analysis of workers' and referents' serum for PCB was much more comprehensive as we quantified 57 congeners compared to 24 (Kontsas) and 19 (Wingfors), our comparison of the congener profiles between US workers and referents documented substantial differences in subject mean serum levels for PCB 6, 16, 26, 33, 37, 41, 70, 97, and 136. In these cases the subject mean exceeded the reference mean by a factor of 5 or more. Most of these congeners (particularly PCB 6, 8, 16, 26, 33, 37, and 41) were not measured in the other studies of workers removing PCB caulk. These congeners found to be most substantially elevated among our subjects are those rapidly eliminated from the body, typically with relative human accumulation (RHA) factors from 0.001 to 0.07 [8] and short apparent human half lives (0.02, years, approximately 1 week) for PCB 33 [1]).

Subject number 6 in our study had the highest serum PCB levels. He is the oldest (at 72), with the longest duration of work with the caulking material (54 years), and he reported extensively installing the PCB caulking material in the 1960s and 70s. While his serum levels were the highest, his congener profile (Figure 3) does not reflect elevations contributed from the lighter PCB congener seen in some other subjects. In fact his light/heavy congener profile as percent contribution to the total is the lowest (4.3%/95.7%) compared to 11.7%/88.4% for the mean of the construction workers, and 6.5%/91.7% for the mean of the referents. His serum level of the lighter congeners (0.378 ng/g, compared to the workers mean of 0.230 ng/g, and referent mean of 0.094 ng/g) was the highest measured, however, even though he had not worked with caulking for over a year preceding the time of sampling.

Subject number 3, on the other hand, had the lowest serum level for the sum of the 57 PCBs, but the greatest contribution to the total from the light congeners (light/heavy distribution of 24.9%/75.1%). The contrast is most striking for congeners 28, 60, and 66, all of which were measured in subject #3 at levels at least twice the mean level of the six construction workers, and up to 50 times the mean level for the referents. Of these three congeners, significant associations were found between airborne exposures and serum PCB 28 levels among the Finnish caulking workers [6], while PCB 56/60 and 66 were reported to be good markers of occupational exposures [7].

## Conclusion

While our study is small, it provides preliminary evidence that workers engaged in the removal of PCB-containing caulk are at risk of PCB exposure, as demonstrated by comparison of their levels, and congener profiles of serum PCB with a referent population of men of similar ages from the same geographic area. As these workers are generally unaware of the risks associated with PCB exposure, or even that they are handling PCB-containing materials, there is a clear need for a requirement that caulking be tested for PCB content before beginning construction work that involves disturbing this material. Further, a larger study to characterize exposure by inhalation, dermal contact and ingestion during caulking removal is needed to develop a control strategy to minimize worker exposure, as well as release of PCB into the environment in and around these buildings.

## Competing interests

The author(s) declare that they have no competing interests.

## Authors' contributions

RFH designed the study and was responsible its overall conduct; JDM and RH were involved in the analysis and interpretation of the data and participated in the manuscript preparation; LA conducted the sample analysis and data compilation; GAW was responsible for recruiting the study subjects and participated in the interpretation of the study results. All authors read and approved the final manuscript.
